# Effects of continuity of care on the postradiotherapy survival of working-age patients with oral cavity cancer: A nationwide population-based cohort study in Taiwan

**DOI:** 10.1371/journal.pone.0225635

**Published:** 2019-12-16

**Authors:** Tsu Jen Kuo, Pei Chen Wu, Pei Ling Tang, Chun-Hao Yin, Chi Hsiang Chu, Yao-Min Hung

**Affiliations:** 1 Department of Stomatology, Kaohsiung Veterans General Hospital, Kaohsiung, Taiwan; 2 Department of Marine Biotechnology and Resources, National Sun Yat-sen University, Kaohsiung, Taiwan; 3 Department of Dental Technology, Shu-Zen junior College of Medicine and Management, Kaohsiung, Taiwan; 4 Department of Molecular Biology and Human Genetics, Tzu Chi University, Hualien, Taiwan; 5 Research Center of Medical Informatics, Kaohsiung Veterans General Hospital, Kaohsiung, Taiwan; 6 Department of Nursing, Meiho University, Pingtung, Taiwan; 7 College of Nursing, Kaohsiung Medical University, Kaohsiung, Taiwan; 8 Clinical Trial Center, Kaohsiung Chang Gung Memorial Hospital, Kaohsiung, Taiwan; 9 Institute of Statistics, National University of Kaohsiung, Kaohsiung, Taiwan; 10 Department of Internal Medicine, Kaohsiung Municipal United Hospital, Kaohsiung, Taiwan; 11 School of Medicine, National Yang Ming University, Taipei, Taiwan; 12 Yuhing Junior College of Health Care and Management, Kaohsiung, Taiwan; University of Wisconsin, UNITED STATES

## Abstract

**Objectives:**

Cancer of the oral cavity, a well-known global health concern, remains one of most common causes of cancer mortality. Continuity of care (COC), a measurement of the extent to which an individual patient receives care from a given provider over a specified period of time, can help cancer survivors process their experiences of dealing with the illness and recuperation; however, limited research has focused on the survival rate of working-age patients with oral cancer.

**Methods:**

A total of 14,240 working-age patients (20 <age ≤65 years) with oral cavity cancer treated with radiotherapy (RT) during 2000–2013 were included in this study from a registry of patients with catastrophic illnesses maintained by the Taiwan National Health Insurance Research Database. We evaluated the effects of the Continuity of Care Index (COCI) proposed by Bice and Boxerman, sociodemographic factors, and comorbidities on the survival rate. This study categorized COC into three groups—low (COCI < 0.23), intermediate (COCI = 0.23–0.37), and high (COCI ≥ 0.38)—according to the distribution of scores in our sample. A multivariate Cox proportional hazards regression model was used to determine the demographic factors and comorbidities associated with the survival rate.

**Results:**

Among all the relevant variables, low COCI, male sex, low socioeconomic status, no receipt of prior dental treatment before RT, residence outside northern Taiwan, chemotherapy receipt, and a history of diabetes increased the risk of mortality. Pre-RT dental evaluation and management was significantly associated with reduced post-RT mortality (adjusted hazard ratio [aHR] = 0.767, 95% confidence interval [CI] = 0.729–0.806, p < 0.001). Compared with patients with a high COCI, those with a low COCI exhibited an increased risk of mortality (aHR = 1.170, 95% CI = 1.093–1.252, p < 0.001). The mortality risk in the intermediate COC group was significantly higher than that in the high COC group (aHR = 1.194, 95% CI = 1.127–1.266, p < 0.001). To balance the distribution of the potential risk factors, propensity-score matching was used for the high COC (COCI > 0.38) and non-high COC (COCI ≤ 0.38) groups. After propensity-score matching, the mortality risk in the low and intermediate COC groups was also found to be significantly higher than that in the high COC group (aHR = 1.178, 95% CI = 1.074–1.292, p < 0.001 and aHR = 1.189, 95% CI = 1.107–1.277, p = 0.001, respectively).

**Conclusions:**

In Taiwan, COC and prior dental treatment before RT significantly affected the survival rate of working-age patients with oral cancer. This result merits policymakers’ attention.

## Introduction

Cancer of the oral cavity, one of the most common malignancies worldwide, is increasingly becoming a global public health concern. Cancer of the oral cavity and oropharynx is the sixth most common cancer worldwide[1, 2]. Approximately 354,864 new diagnoses of oral cancer and 177,384oral-cancer-related deaths were reported in 2018 [3]. Oral cancer is the fifth most common cause of mortality in Taiwan and the fourth most common cause of mortality among Taiwanese men [4, 5]. In addition, an increasing trend has been observed in the incidence of oral cancer among Taiwanese men [6]. Furthermore, oral cancer accounts for the main cause of all malignancies in young adult (25–44 years)patients [7, 8]. According to the Surveillance, Epidemiology, and End Results database, the average diagnosis age of oral cavity cancer in the United States is 63 years[9]. However, we noted that the average diagnosis age of oral cavity cancer in Taiwan is 53 years [10], which is approximately 10 years younger than that in the US population. The mean age at death from oral cavity cancer in Taiwan is 56 ± 13 years. Most patients (72.5%) died between 35 and 64 years of age[5]. Working-age adults (aged20–65 years) are the main source of family income and care. The occurrence of oral cancer in this age group adversely affects the family, society, and country.

Oral cancer has a lower 5-year survival rate (≤50%) than other major types of cancers [11, 12]. The treatment of oral cancer usually depends on the cooperation of a multidisciplinary team and involves surgery, radiotherapy (RT), and/or chemotherapy[9]. RT effectively alleviates oral cancer but may be accompanied by a diverse range of therapy-related side effects, such as radiogenic xerostomia, taste disturbance, opportunistic infections, salivary hypofunction, radiation caries, and progressive periodontal destruction. Schweyen et al. reported that the postradiation oral-health-related quality of life (OHRQoL) is the lowest for oral cancer among all head and neck cancers[13]. Numerous factors affect the survival of patients with oral cancer after RT, including age, sex, race, lifestyle habits (such as smoking, alcohol consumption, betel nut chewing, and physical activities), human papillomavirus status, primary tumor stage, therapy type, nutritional status, psychiatric disorders, urbanization, education, socioeconomic status (SES), and geographical area [14–18].

Continuity of care (COC) is a core element of medical care and represents a trustful and responsible therapeutic relationship between patients and their care providers[19–21]. A long-term physician–patient sustained trustful relationship can enhance mutual communication and effective disease management and improve patient outcomes, particularly for chronic diseases [22, 23]. The Continuity of Care Index (COCI), which was developed by Bice and Boxerman, is a widely accepted measurement tool. The COCI uses the number of physicians providing service to a patient and the percentage of care provided by each physician. This index is generated for each patient and is calculated by dividing the number of visits to each individual physician by the total number of visits by the patient. The COCI measures both the frequency of ambulatory visits to each physician and the dispersion of visits between physicians. The values of the COCI range between 0 (visits made to a number of different physicians) and 1 (all visits made to the same physician).

In cancer care, COC is a major theoretical concept and enhances the quality of care[24, 25]. Cancer and its treatment negatively affect a patient’s psychosocial condition and social life; therefore, promoting COC could help cancer survivors deal with the illness-processing experiences and recuperation [26]. However, according to our review of the relevant literature, no study has yet examined the association between COC and the survival rate in working-age patients with oral cavity cancer. Prognostication of post-RT survival is fundamental for treatment planning by oral reconstruction dentists and counseling patients. Therefore, this study investigated the association of COC, SES (determined by income), and medical comorbidities with post-RT survival among working-age patients with oral cancer in Taiwan.

## Methods

### Data source

This research is a retrospective cohort study based on the National Health Insurance Research Database (NHIRD), a nationwide population-based administrative database that has enrolled almost 99% of the population of Taiwan. The NHIRD has been validated by some independent studies[27, 28]. The advantages of using the NHIRD for research purposes have been previously described [29]. The NHIRD collects beneficiaries’ registration files on demographic data, all types of medical visits, laboratory test codes, procedure codes, prescription codes, and diagnostic codes according to the International Classification of Diseases, 9th Revision, Clinical Modification (ICD-9-CM). This study was approved by the Institutional Review Board of Kaohsiung Veterans General Hospital, Kaohsiung, Taiwan (VGHKS15-EM10-02). Because the data used comprised a deidentified secondary dataset released for research purposes and were analyzed anonymously, the need for informed consent was waived.

### Study cohort

#### Inclusion and exclusion criteria

According to our previous study for the period 2000–2013, the criteria for inclusion in the oral cavity cancer group were diagnosis based on ICD-9 diagnostic codes 140–145, no cancer history, and receipt of RT procedure codes 36012B or 36011B <100 times in 75 days (since RT commenced). The exclusion criteria for the recruitment of patients into the working-age oral cavity cancer group were as follows: (1) age> 65 years or ≤20 years, (2) follow-up period of <0.5 years, (3) incomplete data, and (4) patients with <three outpatient visits during the COC period. Because individuals from the working-age group are the main source of family income and care, the earlier age onset and short expected lifespan (3 years) in cases of oral cavity cancer in Taiwan have a considerably negative effect on the entire society and country [4]. Therefore, we investigated the survival of working-age oral cancer patients who had received a complete course of RT. In total, 14,240 working-age individuals (20 <age ≤65 years) with oral cavity cancer who were treated with RT were included.

#### Study variables

We compared the survival rate among groups with different COCI scores, sociodemographic factors, and medical comorbidities.

#### Measured COCI and COCI groups

Considering the different variations, such as easy medical access, and more frequent physician visits in Taiwan compared with other countries, this study used the COCI developed by Bice and Boxerman to measure the COC [30]. This method is widely accepted in various COC-related studies using health care claim databases [31–34].

The COCI enabled us to obtain the distribution of oral-cancer-related outpatient visits to different physicians and the number of visits to each physician since the index day (the day the head and neck RT began).

According to Bice and Boxerman[30], the COCI is defined as follows:

$$COCI=\frac{\sum_{i=1}^{k}n_{i}^{2}-T}{T(T-1)},$$

where *T* is the total number of oral-cancer-related outpatient visits (oral cancer being the principal diagnosis), *n*_*i*_ is the number of times the patient visited a physician *i*, and *k* is the total number of physicians visited.

In this study, the 3-year COCI was considered to compare the survival times of patients with oral cancer; however, approximately 58.8% of patients had a follow-up period of <3 years (approximately 38.7% died within 3 years). Therefore, we had to adjust the 3-year COCI for analysis.

First, we used the data of the patients with a follow-up period of >3 years (N = 5,895) to fit a linear regression model between 1- and 3-year COCI. The linear regression model is represented as follows:

$${\hat{COCI}}_{3y}=0.065+0.764*{COCI}_{1y},$$

where COCI_1y_ and COCI_3y_ are the 1- and 3-year COCI, with R^2^ being 0.636 (p < 0.001).

**Fig 1:**
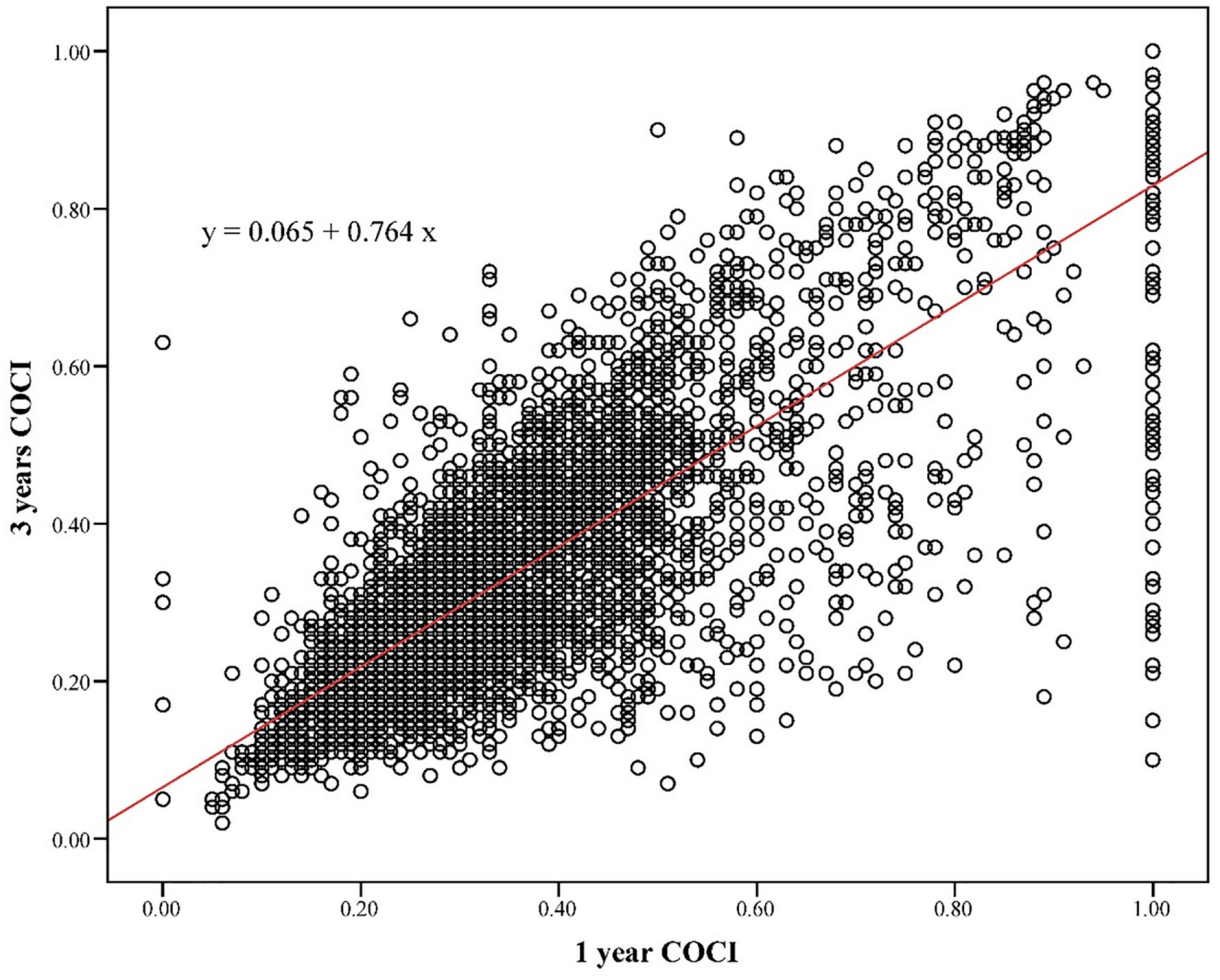
Illustrates the scatter plot between the 1- and 3-year COCI.

Fig 1 illustrates the scatter plot between the 1- and 3-year COCI. We used the aforementioned model to correct the 3-year COCI for the patients with a follow-up period of <3 years.

According to the National Comprehensive Cancer Network guideline for oral cancer, most failures after oral cancer treatment occurred in the first 3 years after treatment. During this period, regular and more intensive oncologist follow-up inspections for usually 1–3 months/time for the first year, 2–4 months/time for the second year, and 4–6 months/time for the third to fifth years are necessary. Most importantly, we selected the 3-year COCI as the endpoint.

#### COCI groups

We used the first and third quantiles of the 3-year COCI (i.e., 0.23 and 0.38, respectively) to divide the COCI groups for analysis. The COCI values ranged from 0 to 1, with a higher value corresponding to a better COC.

### Outcomes and covariates

The outpatient records for each patient in the three COCI groups were tracked from their index day. Other measured variables included sociodemographic factors such as age, sex, urbanization, income, and geographical area; medical comorbidities; and therapy type(therapy used in combination with conventional chemotherapy, pre-RT dental evaluation and management, the year of RT administration, and hospital accreditation level).

The National Health Insurance (NHI) premium status depends on the income of the patients. We used this feature to represent the income factor in our design. We categorized the monthly income as follows: low (<NT$17,500), moderate (NT$17,500–NT$25,000), and high (>NT$25,001). The geographical area was classified as northern, central, southern, and eastern (including the offshore island group) Taiwan. Taiwan has three levels of accreditation for institutions that offer oral cancer therapy, namely medical centers, regional hospitals, and district hospitals. Medical centers have the highest service volume, and regional hospitals have the second highest service volume.

The comorbidities analyzed in this study were hypertension (ICD-9-CM codes 401–405), diabetes mellitus (DM, ICD-9-CM code 250), coronary artery disease (CAD, ICD-9-CM codes 410–414), and depression (ICD-9-CM codes 296.2, 296.3, 300.4, and 311.x).

### Survival analysis

The starting point of survival analysis was the index day, which is defined as the first day of RT. The endpoint of the survival analysis was the day of death. For patients who survived until the end of the observation period, December 31, 2013, was considered the endpoint. All individuals in the three COCI groups were tracked down until death, withdrawal from the NHI program, or the end of 2013, whichever occurred first.

### Statistical analysis

The Pearson chi-square test was used to analyze the categorical variables (sex, geographic region of residence, urbanization, income, medical comorbidities, chemotherapy, and timing of RT), whereas a one-way analysis of variance was employed for assessing the continuous variable (age). The Cox proportional hazards regression model was used to estimate the hazard ratio (HR) of mortality. The candidate variables with *p values* less than 0.05 were selected through univariate analysis, and multivariate analysis was then used to select the important variables of mortality and estimate the adjusted HR (aHR). All statistical analyses were performed using SPSS (version 20; SPSS Inc., Chicago, IL, USA).

## Results

A total of 14,240 working-age patients with oral cavity cancer who were treated with RT (mean age, 49.73 ± 8.20 years; age range, 20.01–65.00 years) were included in the study, and the overall mortality rate (until December 31, 2013) was 48.5%. Using the Kaplan-Meier method, the five-year overall survival rate for oral cavity cancer with RT is 49.3%. The baseline characteristics of the three COCI groups are listed in Table 1. The number of outpatient visits and the distribution (mean, median, and range) of outpatient visits among patients in the low-, intermediate-, and high-COCI groups are presented in Table 1.

**Table 1:** Levels of 3-year COCI by patient characteristics.

Characteristic	Low	Intermediate	High	Total	*p-value*
	(COCI<0.23)	(0.23–0.37)	(COCI≧0.38)		
Cases(%)	3450(24.2%)	7053(49.5%)	3737(26.2%)	14240	
Age, yrs (Mean±SD)	49.80±8.20	49.60±8.28	49.85±8.04	49.73±8.20	0.256
Outpatients visits					<0.001
Mean	86.95±52.84	67.64±35.89	57.84±36.83	69.89±42.27	
Medium	77	61	50	61	
1^st^ - 3^rd^ quantile	51–111	43–85	35–71	41–88	
Physicians					<0.001
Mean	14.95±6.70	9.63±4.61	7.25±4.38	10.33±5.87	
Medium	14	9	6	9	
1^st^ - 3^rd^ quantile	11–18	6–12	4–9	6–13	
Death (until 2013/12/30)	1686 (48.9%)	3428 (48.6%)	1795 (48.0%)	6909 (48.5%)	0.762
Sex (Male)	3244 (94.0%)	6665 (94.5%)	3517 (94.1%)	13426 (94.3%)	0.543
SES (Income)					0.121
High	941 (27.3%)	1836 (26.0%)	944 (25.3%)	3721 (26.1%)	
Medium	1475 (42.8%)	2950 (41.8%)	1602 (42.9%)	6027 (42.3%)	
Low	1034 (30.0%)	2267 (32.1%)	1191 (31.9%)	4492 (31.5%)	
Timing of receiving RT (Before 2009)	1532 (44.4%)	3200 (48.5%)	2334 (62.5%)	7286 (51.2%)	<0.001
Residential area					<0.001
Northern	1028 (29.8%)	2636 (37.4%)	1253 (33.5%)	4917 (34.5%)	
Central	1012 (29.3%)	1877 (26.6%)	1221 (32.7%)	4110 (28.9%)	
Southern	1273 (36.9%)	2554 (32.0%)	999 (26.7%)	4526 (31.8%)	
Eastern	137 (4.0%)	286 (4.1%)	264 (7.1%)	687 (4.8%)	
Urbanization level					<0.001
Urban	780 (22.6%)	1806 (25.6%)	829 (22.2%)	3415 (24.0%)	
Suburban	1671 (48.5%)	3391 (48.1%)	1774 (47.5%)	6836 (48.0%)	
Rural	997 (28.9%)	1854 (26.3%)	1133 (30.3%)	3984 (28.0%)	
Hospital accreditation level					<0.001
Medical center	2492 (72.2%)	5143 (72.9%)	2651 (70.9%)	10286 (72.2%)	<0.001
Regional hospital	944 (27.4%)	1869 (26.5%)	1033 (27.6%)	3846 (27.0%)	
District hospital	14 (0.4%)	41 (0.6%)	53 (1.4%)	108 (0.8%)	
Pre-RT dental evaluation and management Yes	2626 (76.1%)	5034 (71.4%)	2338 (62.6%)	9998 (70.2%)	<0.001
Combine chemotherapy	2534 (73.4%)	5004 (70.9%)	2221 (59.4%)	9759 (68.5%)	<0.001
Comorbidity					
DM (%)	661 (19.2%)	1179 (16.7%)	546 (14.6%)	2386 (16.8%)	<0.001
H/T (%)	1042 (30.2%)	1950 (27.6%)	847 (22.7%)	3839 (27.0%)	<0.001
CAD (%)	319 (9.2%)	531 (7.5%)	230 (6.2%)	1080 (7.6%)	<0.001
Depression (%)	176 (5.1%)	236 (3.3%)	101 (2.7%)	513 (3.6%)	<0.001

Abbreviations: COCI, Continuity of Care Index; SES, socioeconomic status; RT, radiotherapy; DM, diabetes mellitus; H/T, hypertension; CAD, coronary artery disease.

Among all the relevant variables, male sex, residential area, income, comorbidity of diabetes, year of RT, hospital accreditation level, pre-RT dental evaluation and management, COCI, and use of combined chemotherapy were associated with the mortality of patients with oral cancer receiving RT (Table 2).

**Table 2:** Univariate and multivariate Cox proportional hazard models for survival analysis.

Variables	Total cases	No. of events	Univariate		Multivariate	
			HR(95%CI)	*p-value*	Adj. HR(95%CI)	*p-value*
Age			1.000(0.997–1.002)	0.763		
Sex (Male)	13426	6600	1.442(1.287–1.617)	<0.001	1.425(1.271–1.598)	<0.001
SES(Income)						
High	3721	1682	Reference		Reference	
Medium	6027	2930	1.129(1.063–1.199)	<0.001	1.111(1.046–1.180)	0.001
Low	4492	2297	1.250(1.174–1.331)	<0.001	1.236(1.160–1.316)	<0.001
Timing of receiving RT Before 2009	7286	4483	1.244(1.182–1.310)	<0.001	1.292(1.225–1.363)	<0.001
Residential area						
Northern	4917	2192	Reference		Reference	
Central	4110	2008	1.075(1.012–1.142)	0.019	1.105(1.039–1.174)	0.001
Southern	4526	2341	1.307(1.233–1.385)	<0.001	1.356(1.278–1.439)	<0.001
Eastern	687	368	1.263(1.131–1.410)	<0.001	1.308(1.171–1.462)	<0.001
Urbanization level						
Urban	3415	1609	Reference	0.193		
Suburban	6836	3341	1.050(0.990–1.115)	0.106		
Rural	3984	1959	1.057(0.989–1.129)	0.102		
Hospital accreditation level						
Medical center	10286	5027	Reference		Reference	
Regional hospital	3846	1800	1.024(0.970–1.080)	0.398	1.010(0.956–1.067)	0.723
District hospital	108	82	2.068(1.662–2.572)	<0.001	1.892(1.519–2.357)	<0.001
Pre-RT dental evaluation and management	9998	4407	0.736(0.701–0.773)	<0.001	0.767(0.729–0.806)	<0.001
Combine chemotherapy	9759	5016	1.579(1.498–1.666)	<0.001	1.621(1.536–1.710)	<0.001
Comorbidity						
DM	2386	1177	1.090(1.023–1.160)	0.007	1.120(1.052–1.193)	<0.001
H/T	3839	1776	1.009(0.956–1.065)	0.742		
CAD	1080	513	1.015(0.927–1.110)	0.749		
Depression	513	248	1.110(0.978–1.260)	0.107		
3 year COCI High (≧0.38)	3737	1795	Reference		Reference	
Intermediate	7053	3428	1.173(1.097–1.254)	<0.001	1.194(1.127–1.266)	<0.001
Low (<0.23)	3450	1686	1.193(1.126–1.263)	<0.001	1.170(1.093–1.252)	<0.001

Abbreviations: COCI, Continuity of Care Index; SES, socioeconomic status; RT, radiotherapy; DM, diabetes mellitus; H/T, hypertension; CAD, coronary artery disease.

### Propensity-score matching

Because the different COCI groups exhibited different ratios of hospital accreditation level and comorbidities, which may have influenced the mortality risk, we performed propensity-score matching to balance the distribution of these potential risk factors. After propensity-score matching, no significant difference was observed in the mortality risk between the two groups (COCI <0.38 [N = 3737] vs. COCI≥ 0.38 [N = 3737]; Table 3).

**Table 3:** Characteristics of matching variables for matching paired patients (propensity score).

Variables	COCI <0.38	COCI≧0.38	*p-value*
	(n = 3737)	(n = 3737)	
Age, years (Mean±SD)	49.82±8.31	49.85±8.04	0.875
Sex (Male)	3510 (93.9%)	3517 (94.1%)	0.767
Residential area			0.747
Northern	1286 (34.4%)	1253 (33.5%)	
Central	1238 (33.1)	1221 (32.7%)	
Southern	962 (25.7%)	999 (26.7%)	
Eastern	251 (6.7%)	264 (7.1%)	
SES(Income)			0.955
High	1201 (32.1%)	1191 (31.9%)	
Medium	1578 (42.2%)	1602 (42.9%)	
Low	958 (25.6%)	944 (25.3%)	
Timing of receiving RT Before 2009	2336 (62.5%)	2334 (62.5%)	0.975
Hospital accreditation level			0.169
Medical center	2672 (71.5%)	2651 (70.9%)	
Regional hospital	1023 (27.4%)	1033 (27.6%)	
District hospital	42 (1.1%)	53 (1.4%)	
Pre-RT dental evaluation and management	2339 (62.6%)	2338 (62.6%)	1.000
Combine chemotherapy	2209 (59.1%)	2221 (59.4%)	0.750
DM	537 (14.4%)	546 (14.6%)	0.789

Abbreviations: COCI, Continuity of Care Index; SES, socioeconomic status; RT, radiotherapy; DM, diabetes mellitus.

Finally, we used the multivariable Cox proportional hazards model for survival analysis among propensity-score-matched patients, and the results are presented in Table 4. After adjustment for other confounding factors, the adjusted risk for patients in the low-COCI group was significantly higher than that for those in the high-COCI group (aHR = 1.178(1.074–1.292); 95% confidence interval [CI] = 1.074–1.292, p = 0.001). The aHR (95% CI) in the intermediate-COCI group was significantly higher than that in the high-COCI group. Thus, COCI was an independent prognostic factor for oral cavity cancer survival.

**Table 4:** Univariate and multivariate Cox proportional hazard models for survival analysis among propensity-score-matched patients.

Variables	Total cases	No. of events	Univariate		Multivariate	
			HR(95%CI)	*p-value*	Adj.HR(95%CI)	*p-value*
Age			1.001(0.997–1.005)	0.685		
Sex (Male)	7027	3561	1.483(1.270–1.731)	<0.001	1.485(1.272–1.734)	<0.001
SES(Income)						
High	1902	855	Reference		Reference	
Medium	3180	1609	1.202(1.106–1.306)	<0.001	1.186(1.091–1.289)	<0.001
Low	2392	1265	1.325(1.124–1.445)	<0.001	1.293(1.185–1.410)	<0.001
Timing of receiving R/T Before 2009	4670	2842	1.371(1.268–1.482)	<0.001	1.375(1.271–1.488)	<0.001
Residential area						
Northern	2539	1177	Reference		Reference	
Central	2459	1248	1.077 (0.995–1.167)	0.066	1.095(1.010–1.187)	0.027
Southern	1961	1027	1.246(1.146–1.355)	<0.001	1.293(1.187–1.408)	<0.001
Eastern	515	277	1.209(1.061–1.379)	<0.001	1.221(1.071–1.393)	0.003
Hospital accreditation level						
Medical center	5323	2681	Reference		Reference	
Regional hospital	2056	976	1.024(0.952–1.102)	0.525	0.998(0.926–1.075)	0.956
District hospital	95	72	2.196(1.737–2.775)	<0.001	1.975(1.559–2.503)	<0.001
Pre-RT dental evaluation and management	4677	2060	0.693(0.650–0.739)	<0.001	0.734(0.688–0.784)	<0.001
Combine chemotherapy	4430	2387	1.577(1.475–1.687)	<0.001	1.614(1.508–1.727)	<0.001
Comorbidity						
DM	1083	547	1.075(0.981–1.177)	0.121		
H/T	1862	874	0.993(0.920–1.071)	0.857		
CAD	531	257	0.999(0.880–1.134)	0.983		
Depression	240	117	1.101(0.916–1.323)	0.370		
3 year COCI						
High (≧0.38)	3737	1795	Reference		Reference	
Intermediate	2592	1330	1.175(1.095–1.262)	<0.001	1.189(1.107–1.277)	<0.001
Low (<0.23)	1145	604	1.166(1.064–1.279)	0.001	1.178(1.074–1.292)	0.001

Abbreviations: COCI, Continuity of Care Index; SES, socioeconomic status; RT, radiotherapy; DM, diabetes mellitus; H/T, hypertension; CAD, coronary artery disease.

District hospitals were the most significantly associated with reduced post-RT survival of patients with oral cancer [aHR = 1.892, 95% confidence interval (CI), 1.519–2.357, p < 0.001], whereas pre-RT dental evaluation and management was significantly associated with reduced post-RT mortality (aHR = 0.767, 95% CI = 0.729–0.806, p < 0.001). Men had a greater risk of mortality than women (aHR = 1.425, 95% CI = 1.271–1.598, p < 0.001). The aHR (95% CI) for patients with low income compared with those with high income was1.236 (1.160–1.316), whereas the aHR (95% CI) for patients with medium income compared with those with high income was 1.111 (1.046–1.180).

Compared with patients residing in northern Taiwan, those residing in central, southern, and eastern Taiwan had 10.5%, 35.6%, and 30.8% greater risk of mortality, respectively (p ≤ 0.001). Patients with a history of diabetes exhibited significant risk factors of mortality among working-age patients with oral cancer in Taiwan (aHR = 1.120, 95% CI = 1.052–1.193, p < 0.001). Regarding the hospital accreditation level, compared with medical centers, regional hospitals and district hospitals represented a 1% and 89.2% greater risk of mortality, respectively. The aHR (95% CI) for patients who received a combination treatment of chemotherapy compared with that of patients who did not receive chemotherapywas1.621 (1.536–1.710) (p < 0.001). The aHR (95% CI) for patients who received RT the year before 2009 compared with those patients who received RT in 2009 or after was 1.292 (1.225–1.363) (p < 0.001). The post-RT association of the 3-year COCI and mortality of working-age patients with oral cancer is presented in Table 2. Among the various subgroups of the 3-year COCI, patients in the highest COCI group had the lowest mortality rate.

After adjustment for other confounding factors, the adjusted risk for patients in the low-COCI group was significantly higher than that for those in the high-COCI group (aHR = 1.170; 95% CI = 1.093–1.252, p < 0.001). The aHR (95% CI) in the intermediate-COCI group was significantly higher than in the high-COCI group (aHR = 1.1924; 95% CI 1.127–1.266, p < 0.001). However, no significant difference was found between the intermediate-and low-COCI groups.

### Sensitivity analysis using different COCI cutoffs

To determine whether the significance of the COCI between groups is dependent on the selected cutoffs, we used various COC cutoffs. We used binary groupings for the analysis, that is, we defined high- and low-COCI groups on the basis of different cutoffs ranging from 0.32 to 0.50. Table 5 presents the results of the multivariate Cox regression analysis obtained using different COCI cutoffs in the study cohort. The COCI was an independent prognostic factor for oral cavity cancer survival.

**Table 5:** Multivariate Cox proportional hazard models for survival analysis at different COCI cutoffs.

COCI Cut point	% of high COC	Adj.HR(low vs high COC)	*p-value*	95% C.I.
0.32	39.0%	1.115	<0.001	1.062–1.172
0.34	34.0%	1.105	<0.001	1.050–1.162
0.36	28.9%	1.139	<0.001	1.080–1.202
0.38	25.6%	1.141	<0.001	1.080–1.206
0.40	21.9%	1.173	<0.001	1.106–1.243
0.42	18.2%	1.181	<0.001	1.110–1.257
0.44	15.8%	1.197	<0.001	1.121–1.279
0.46	13.0%	1.196	<0.001	1.114–1.283
0.48	11.4%	1.186	<0.001	1.101–1.278
0.50	9.8%	1.197	<0.001	1.105–1.296

Abbreviations: COCI, Continuity of Care Index

We used 10-fold cross-validation to verify the models. All samples with at least a 3-year survival were randomly divided into 10 disjoint sets, of which nine sets were sequentially used to construct the model and one set was used to test the model at each time point (10 repeats). Table 6 indicates that the R^2^ of the training and testing models was 0.636 and 0.634, respectively.

**Table 6:** A second COC model.

Model	Training	Testing
Min. R^2^	0.630	0.568
Max. R^2^	0.643	0.682
Mean R^2^	0.636	0.634
Min. RMSE	0.103	0.098
Max. RMSE	0.104	0.108
Mean RMSE	0.103	0.103

Abbreviations: RMSE, root-mean-square error

## Discussion

To the best of our knowledge, this research is the first nationwide population-based cohort study to demonstrate that low COCI and no pre-RT dental evaluation and management are associated with mortality in working-age patients with oral cavity cancer.

This study has some strengths. First, this research is the first large cohort study to investigate the association between COC and the subsequent mortality in working-age patients with oral cavity cancer. Taiwan’s NHIRD is one of the largest nationwide population databases in the world, covering approximately 23 million residents in Taiwan. The study cohorts were sufficiently large for the analysis of risk variations among subgroups. Second, we identified that the high-risk groups among working-age patients with oral cavity cancer, such as male patients, patients without prior dental evaluation and management, patients who received a combination chemotherapy, patients treated at a hospital with a low accreditation level, and patients with a history of diabetes, had an increased risk of mortality. Third, we used two additional statistical methods, namely propensity-score weighting and sensitivity analysis, to increase the robustness of our finding that COC is associated with the survival of working-age patients with oral cavity cancer in Taiwan.

In the present study, COC was defined as adherence to multidisciplinary management from the time of commencement of RT for oral cancer. Our results suggested that the COC level remained an independent risk factor for oral cavity cancer post RT mortality after adjustment for sex, age, SES, comorbidities, and hospital accreditation level. COC affects the therapeutic relationship, mutual trust, understanding, and communication quality between patients and health care providers, and it improves medication adherence and patient compliance[32, 35]. Frenkel et al. reported that the relationship between health care providers and cancer patients significantly affects survival in cancer[36], and COC can be influenced or modified more easily than personal and clinical factors or some other unchangeable factors (e.g., income, age, cancer type, or cancer stage)[37]. COC plays a major role in optimizing outcomes in cancer care[38, 39]. In the present study, a higher COC level was associated with higher survival in patients with oral cancer.

The Taiwan NHI (TNHI) scheme is a unique health plan. This universal health care scheme ensures that every Taiwanese person has free access to comprehensive medical care. The comprehensive coverage includes inpatient admission, emergency service and outpatient care, prescription drugs, traditional Chinese medicine, dental services, and home nursing care. Because the plan covers more than 99.6% of Taiwanese residents and because almost 92% of different types of hospitals or medical/dental clinics have signed contracts with the Bureau of National Health Insurance, Taiwanese patients have a wide choice of physicians or hospitals at various locations in Taiwan without any barriers. In addition, the waiting times are short. The covered patients can directly go to any specialty care center without a referral. Each TNHI user holds a health insurance card that contains the user’s medical data, including the catastrophic illnesses certificate, prescription drugs information, and some laboratory data. When the covered patients visit physicians(e.g., general practitioners or specialists), the physicians can instantly provide diagnoses to patients. Therefore, cancer patients in Taiwan can choose to visit several oncology centers to get the treatment. Therefore, we designed this study to evaluate the effects of COCI on oral cavity cancer survival. By contrast, in other regions of the world (such as the United States), patients tend to have a single physician per oncology specialty. However, under the TNHI scheme, patients can obtain direct free access to multiple physicians per oncology specialty. This situation may result in disorganized and uncoordinated care. Therefore, a high COCI represents better care coordination and reduced fragmentation across health care providers.

Numerous studies have demonstrated that higher level of SES is associated with higher survival rate among patients with oral cancer [16, 40, 41]. In the present study, low income had a significantly negative association with the survival of patients with oral cancer who were treated with RT. Compared with patients residing in northern Taiwan, those residing in other geographical areas had a greater risk of mortality. This result may reflect the greater availability of medical care in northern Taiwan [42]. The result also highlights the need for improvement in the inequality in regional medical access within Taiwan health literacy and behavior among poorer populations should be improved to reduce the mortality caused by oral cancer. In Taiwan, nearly 10% of the population (approximately 2 million people) has the habit of chewing betel nut, accounting for 16.5% of the male population and 2.9% of the female population [43]. Most of this population is characterized by low socioeconomic status, smoking habits, drinking habits, blue-collar classes, and aborigines [44]. According to the Taiwan epidemiological study published by Professor Kao of the Kaohsiung Medical University in 1995, the incidence of oral cancer was 123-fold higher in patients who smoked, drank alcohol, and chewed betel quid than in abstainers[45]. The proportion of people with the habit of betel nut chewing remains high in Taiwan, and the Taiwanese government should implement more effective measures for public awareness regarding the prevention of oral cancer, thereby addressing the problem of health care inequality.

Apart from low SES, patients with DM exhibited an increased risk of mortality. Hyperglycemia was associated with carcinogenesis and cancer metastasis in patients with type 2 diabetes [46]. According to Gong’s meta-analyasis research, type 2DM is associated with an increased mortality in patients with oral cancer [47].

Another notable finding is that patients with pre-RT dental evaluation and management had a lower risk of mortality than patients who did not receive the evaluation and management. Patients undergoing head and neck RT are at a risk of multiple oral complications, which significantly affects morbidity and reduces OHRQoL among these patients [48, 49]. In addition to the regular oncology management plan, dental assessment before head and neck cancer treatment should include a comprehensive evaluation of dental, medical, social, and environmental factors. The aim should be to establish the risk of oral diseases, identify and plan for the removal of infection foci, prepare the patient for the side effects of oncology treatments, and implement preventative plans for the increased oral disease during/after RT[50].

Adequate pre-RT dental evaluation and management could optimize the oral health status, minimize the complications, and reduce the adverse effects of RT[48, 51, 52]. In the present study, pre-RT dental evaluation and management was associated with a reduced mortality risk. The clinical practice of pre-RT dental evaluation and management involves the comprehensive cooperation of physicians and patients. Before RT, clinicians must not only focus on the medical aspects of oral cancer but also consult the oral surgeon or specialist dentist to record the dental condition of the patient and for pre-RT dental management. Because RT for oral cancer is usually complicated with many oral and dental complications (such as mucositis, dysgeusia, opportunistic infections, xerostomia and salivary hypofunction, trismus, radiogenic dental caries, teeth demineralization, osteoradionecrosis, and progressive periodontal destruction), the oral surgeon or specialist dentist may need to remove the hopeless teeth to avoid potential oral infection. However, knowing more about the prognosis is critical for counseling patients and recommending dental reconstruction. Patients with oral cancer should be aware of proper oral hygiene and possible oral and dental complications. After the initial head and neck RT, a 3-month regular dental follow-up for oral examination, dental scaling, and professional fluoride application are recommended for oral cancer patients. In brief, pre-RT dental evaluation could potentially be a surrogate for a higher level of multidisciplinary care.

Intensity-modulated RT (IMRT) and volumetric-modulated arc therapy(VMAT)outperform conventional RT and three-dimensional conformal RT with higher target coverage, greater efficiency, fewer complications, shorter therapy duration, and less influence on the quality of life. Conventional RT and three-dimensional conformal RT have been replaced by IMRT and VMAT since 2009 in Taiwan[53]. Therefore, the cutoff point in the present study was 2009.

The findings from our study have both clinical and public health implications. Clinically, when treating working-age patients with oral cancer, clinicians must focus not only on the medical aspects of oral cancer but also on pre-RT dental evaluation and management, counseling patients on the importance of COC, comorbidities, and SES status. Patients with DM are at a high risk of mortality and should be referred to adequate specialists. Moreover, all working-age patients with oral cancer should be referred to a dentist for evaluation before RT. From a public health perspective, policymakers are encouraged to enforce DM screening in working-age patients with oral cancer and to provide more COC and integrated care, such as pre-RT dental care.

Several limitations should be considered when interpreting our findings. First, information on potential confounding factors, such as the tumor node metastasis stage, nutritional status, human papilloma virus status, education level, life quality, inherited defects (gene mutations), alcohol drinking habits, smoking history, and betel quid chewing behaviors and habits, was unavailable. Although ICD-9 includes the diagnosis of alcohol and tobacco abuse, the codes for these conditions were seldom used in the NHIRD and are unreliable. In previous NHIRD studies, information on the condition of substance abuse was collected through face-to-face interviews[54]. We did not use the ICD-9 codes to identify patients with an established diagnosis of alcohol use disorder because patients who consumed light or moderate amounts of alcohol or who were not evaluated by physicians were not recorded in the NHIRD[55]. The NHIRD is primarily a health insurance database and contains limited information on alcohol use[56]. Second, the RT protocol type (i.e., conventional RT, three-dimensional conformal RT, IMRT, or VMAT), either palliative or curative, also affects the survival rate of patients and was not available for inclusion in the analysis. New RT techniques, such as IMRT and VMAT, may not have been simultaneously introduced in all four geographical areas of Taiwan. This uncontrolled bias might confound the higher mortality discovered in eastern Taiwan [16]. Third, we eliminated patients with missing data; however, we had to approximate the value of the 3-year COCI for almost 40% of patients, which is a significant limitation. According to aforementioned results and COCI definition, fragmented or uncoordinated clinical care could not be distinguished from necessary multi-discipline care.

## Conclusions

This 14-year population-based cohort study demonstrated an elevated risk of mortality in working-age patients with oral cancer with low COC, low SES, and no pre-RT dental evaluation and management who received treatment in district hospitals, residence outside northern Taiwan, and had a history of diabetes. Monitoring these groups of patients who may be at a high risk of mortality is crucial.
